# Construction of tissue engineered cornea with skin-derived corneal endothelial-like cell and mechanism research for the cell differentiation

**DOI:** 10.3389/fmed.2024.1448248

**Published:** 2024-09-02

**Authors:** Lin Shen, Fang Han, Lijie Pan, Liqun Du, Peng Sun, Kai Zhang, Xinyi Wu, Kunpeng Pang, Jing Zhu

**Affiliations:** ^1^Department of Ophthalmology, Qilu Hospital of Shandong University, Jinan, China; ^2^Beijing Institute of Ophthalmology, Beijing Tongren Eye Center, Beijing Tongren Hospital, Capital Medical University, Beijing, China; ^3^Department of Ophthalmology, The Affiliated Yantai Yuhuangding Hospital of Qingdao University, Yantai, China; ^4^Department of Ophthalmology, Shandong Second Provincial General Hospital, Jinan, China

**Keywords:** corneal endothelial cell-like cells, skin-derived precursors (SKPs), cell differentiation, signal pathway, tissue-engineered cornea (TEC)

## Abstract

**Introduction:**

Corneal endothelial transplantation accounts for most of corneal transplantation for treating corneal diseases, however severe shortage of corneal donors is the biggest obstacle. In our previous study, we differentiated human skin-derived precursors (SKPs) into corneal endothelial cell (CEC)-like cells with a co-culture system. In this study, we aimed to investigate cell differentiation molecular mechanism and evaluate the function of CEC-like cells by developing tissue-engineered corneas in order to improve cell production efficiency and provide basic research for clinical transformation.

**Methods:**

We performed transcriptome sequencing of SKPs and CEC-like cells. Further, we focused on the possible enriching pathways, including PI3K/Akt, MAPK/Erk, WNT/β-catenin, and important transcription factors Pitx2 and Foxc1. The PI3K and β-catenin inhibitors were also added to the culture system to observe the differentiation alteration. We developed a graft for a tissue-engineered cornea (TEC) using CEC-like cells and acellular porcine cornea matrix scaffold. The tissue-engineered corneas were transplanted into rabbits via penetrating keratoplasty.

**Results:**

The PI3K/Akt, MAPK/Erk, and WNT/β-catenin pathways play important roles during the differentiation of SKPs into CEC-like cells. Crosstalk existed between the PI3K/Akt and MAPK/Erk pathways. The PI3K/Akt and WNT/β-catenin pathways were connected. Pitx2 and Foxc1 were subject to temporal and spatial controls of the WNT/β-catenin pathway. The inhibition of the PI3K/Akt and WNT/β-catenin pathways both prevented cell differentiation. CEC-like cells grew well on the acellular porcine cornea matrix scaffold, and the tissue-engineered corneal graft performed well after transplantation into rabbits.

**Conclusion:**

We provide experimental basis for CEC-like cell industrial production and drive the cells to be clinically applied in cellular replacement therapy or alternative graft substitution for treating corneal diseases in the future.

## 1 Introduction

The cornea, recognized as the eye’s foremost transparent and blood-vessel-free tissue, houses corneal endothelial cells (CECs) in its innermost layer (1). These monolayer cells, characterized by their distinctive hexagonal shape, create an obstacle between the corneal stroma and the aqueous fluid by forming occluding junctions. Ionic pumps are important for maintaining corneal transparency (2). Corneal blindness is the principal cause of visual deficiency. Trauma, ulcerations, and Fuchs’ corneal endothelial dystrophy are major causes of corneal blindness. Corneal blindness is exclusively treated via corneal grafting techniques including penetrating keratoplasty, lamellar keratoplasty, and corneal endothelial keratoplasty. Among these, endothelial keratoplasty represents about 39% of all corneal transplant procedures (3). Nonetheless, a worldwide scarcity of corneal donors presents a significant hurdle to performing corneal transplants. As a result, the quest for novel, viable sources of corneal endothelial cells has surfaced as a pressing need in recent years (4).

Our team has engineered CEC-like cells from human SKPs utilizing a three-dimensional co-culture system. These cells exhibit morphology, characteristics, and functions akin to those of human CECs (5). Moreover, in a monkey model of endothelial dysfunction, these CEC-like cells have shown the ability to acclimate to the microenvironment while preserving their key features (6). However, these cells faced constraints in expansion and passage, highlighting the need to delve into the molecular mechanisms underlying these processes. SKPs, being multipotent and related to embryonic neural crest precursors, exhibit a number of similarities with neural crest (NC) stem cells (7). The corneal endothelium originates from the periocular mesenchyme (POMP), essentially meaning it derives from periocular neural crest (NC) cells (8, 9). Hence, we theorize that the differentiation process we employed likely mimics the *in vitro* transformation of NC cells into endothelial cells. Therefore, we aimed to elucidate the mechanism of CEC-like cell differentiation from SKPs in order to improve cell production efficiency and provide basic research for clinical transformation.

The progression of TECs is a superb substitute for corneal graft substitution and has been a research hotspot globally, especially within the recent twenty years (10, 11). TECs are composed of seed cells and scaffold materials. Scientists have tried several materials for use as scaffolds; these include collagen, silk fibroin film, gelatin, acellular porcine cornea matrix (APCM), and acellular human cornea matrix. They have also seeded cells with rabbit, porcine, bovine, and human corneal cells to develop tissue-engineered anterior and posterior corneal lamella or a full-thickness cornea (12–19). Scaffold material serves as a carrier to support cell adhesion and growth. The APCM and human cornea have similar biological properties, such as tissue structure and mechanical strength. The APCM also has good biocompatibility and low immunogenicity (20–22). These make APCM an ideal scaffold material for TECs. Penetrating keratoplasty remains the most common procedure for treating corneal diseases. In prior research, we administered treatments for corneal endothelial dysfunction through anterior chamber injections. Consequently, it becomes imperative to investigate the use of CEC-like cells as foundational cells for creating tissue-engineered corneas, aiming for broader applications.

In this research, we conducted a comprehensive analysis of the molecular processes involved in the specialization of CEC-like cells from SKPs. We carried out transcriptome sequencing and detailed research on the possible enriching pathways, including PI3K/Akt, MAPK/Erk, and WNT/β-catenin and the important transcription factors Pitx2 and Foxc1. We have shown the activity and complicated relationships between these pathways and factors during corneal endothelial cell differentiation. To delve deeper into the capabilities and uses of CEC-like cells, we crafted a tissue-engineered cornea utilizing these cells. The CEC-like cells exhibited robust growth on the APCM scaffold, and the tissue-engineered cornea graft showed positive outcomes in rabbit models. We provide experimental basis for CEC-like cell industrial production and drive the cells to be clinically applied in cellular replacement therapy or alternative graft substitution for treating corneal diseases in the future.

## 2 Methods

### 2.1 Cell culture

Human SKPs were obtained and cultivated following earlier established methods (23, 24). Skin samples were collected from 10 individuals (average age: 32.9 ± 15.82 years) undergoing eyelid surgeries. In summary, the freshly obtained human eyelid skin was treated with dispase II (Sigma) at 4°C for 12–24 h. Subsequently, the epidermal layer was separated, and the underlying dermal tissue was finely chopped and then digested in type I collagenase (Sigma) at 37°C for 1–3 h. The culture medium for the SKPs included DMEM/F12 Nutrient Mixture (3,1 ratio; Thermo Fisher) enhanced with 1% penicillin/streptomycin (Solarbio), 2% B27 supplement (Thermo Fisher), 40 ng/mL human recombinant bFGF, and 20 ng/mL EGF (both sourced from Peprotech). This medium was refreshed every 4th to 5th day, and the SKP spheres underwent passage at intervals of 7 to 10 days.

The immortalized HCEC cell line B4G12 was kindly supplied by Monika Valtink and cultivated following established protocols (25). In summary, B4G12 cells were grown in human endothelial-SFM medium (Thermo Fisher) enriched with 10 ng/mL human recombinant bFGF and 1% penicillin/streptomycin.

### 2.2 Differentiation of SKPs into CEC-like cells

CEC-like cells were generated from human SKPs following the protocol we previously established (5). In a concise manner, individual cells were obtained from human SKP spheres using 0.05% trypsin and 0.02% EDTA solution, after which they were seeded onto 6-well plates previously covered with 10 mg/mL chondroitin sulfate and 10 μg/mL laminin (sourced from Sigma) at a concentration of 5 × 10^5^ cells/mL. To produce CEC-like cells, non-contact co-culture was employed using transwells (Corning), with B4G12 cells situated in the top compartment and SKP cells positioned in the lower compartment, all within B4G12 culture medium. The medium was refreshed every 2 days. Cultivation of all cells occurred in a moist incubator maintained at 37°C and 5% CO_2_.

### 2.3 RNA-sequencing of SKPs and CEC-like cells

Total RNA from SKPs and CEC-like cells was extracted utilizing TRIzol reagent (Life Technologies), adhering to the instructions provided by the manufacturer. A transcriptome library for RNA sequencing was constructed using the TruSeq^™^ RNA Sample Preparation Kit from Illumina (San Diego, CA), starting with 1 μg of total RNA. Sequence analysis took place at Majorbio Biotech (Shanghai, China) on the Illumina HiSeq 4,000 platform, employing 150 bp paired-end reads. The initial paired-end reads underwent trimming and quality checks using SeqPrep and Sickle with standard settings. Subsequently, the purified reads were matched to the human reference DNA sequence in an orientation-aware manner utilizing the HISAT software. For each sample, aligned reads were then compiled using StringTie.

To detect differentially expressed genes (DEGs) across two distinct samples, their expression levels were determined using the TPM (Transcripts Per Million) method. Gene abundances were quantified by RSEM. For the analysis of differential expression, the R statistical package EdgeR was employed. Genes were deemed significantly differentially expressed if they had an FDR (False Discovery Rate) < 0.05 and an absolute log2 fold change (|log2FC|) of at least 1. Venn diagrams (for TPM > 1) illustrated the overlap and distinct gene expressions among the specimens. This association study serves as a foundational reference for examining differential genes. Functional enrichment analysis of DEGs was conducted using Goatools, identifying markedly enhanced genes at a Bonferroni-corrected *p*-value ≤0.05 against the backdrop of the entire transcriptome.

### 2.4 Immunofluorescent staining

Immunofluorescence staining was performed using established protocols. In brief, cells or tissue samples were preserved in 4% paraformaldehyde for 10 min and subsequently obstructed with 5% goat serum albumin for 1 hour. Overnight incubation with primary antibodies was done at 4°C. For visualization, secondary antibodies (1:100, Huabio, HA1122 and HA1126) were applied, and nuclei were highlighted with DAPI staining (Solarbio). A fluorescent microscope was utilized for examining fluorescence. The primary antibodies used included Na^+^/K^+^ ATPase (1:100, Novus, NB300-146), ZO-1 (1:100, CST, 13663S), PITX2 (1:100, Abcam, ab98297), and human nuclei (1,100, Millipore, MAB1281).

### 2.5 Real-time reverse transcription polymerase chain reaction

Total RNA was extracted from SKPs and CEC-like cells using Trizol Reagent (Invitrogen) according to the manufacturer’s protocol. 1 μg sample of total RNA was reverse-transcribed to cDNA (complimentary DNA) with the ReverTra Ace a kit (Toyobo). qRT-PCR analysis was performed using the SYBR Green enzyme mixture (Toyobo) and the Applied Roche 480 Real-Time PCR system according to the manufacturer’s protocol.

### 2.6 Western blotting

Western blot analysis was conducted using conventional techniques. In summary, total proteins from SKPs and CEC-like cells were isolated utilizing a protein extraction kit (Solarbio) and their concentrations determined via the bicinchoninic acid protein assay kit (Beyotime). The proteins underwent separation through 8% SDS-PAGE and were subsequently moved onto polyvinylidene fluoride membranes (Millipore). The membranes were treated with 5% nonfat milk at ambient temperature for 2 h, then incubated overnight at 4°C with appropriately diluted primary antibodies. The primary antibodies targeted were Na^+^/K^+^ ATPase (1:2500, Novus, NB300-146), PITX2 (1:1000, Abcam, ab98297), FOXC1 (1:1000, Abcam, ab223850), P-AKT (1:2000, CST, 4060 T), AKT (1:1000, CST, 4691 T), P-ERK (1:2000, CST, 4370 T), ERK (1:1000, CST, 4695 T), p-GSK-3β (1:1000, CST, 5558 T), GSK-3β (1:1000, CST, 12456 T), active β-catenin (1:1000, CST, 8814 T), β-catenin (1:1000, CST, 8480 T), and β-actin (1:1000, ZSGB-BIO, TA-09). Post immunoblotting with HRP-linked secondary antibodies (1:5000, Beyotime, A0208 and A0216) for 1 h at ambient temperature, protein bands were visualized utilizing a boosted chemiluminescence agent (PerkinElmer) and detected utilizing an automated chemiluminescence imaging scanner (Tanon). Relative protein levels were analyzed using Image J software.

### 2.7 Inhibition of PI3K/Akt and WNT/β-catenin pathways during cell induction

Upon SKPs were incubated together with B4G12 cells in transwell systems, inhibitors for PI3K (LY294002 at 10 μM, Selleck) and β-catenin (XAV-939 at 1 μM, Selleck) were introduced into the induction environment independently. Observations were made regarding the morphology of the cells thus induced. Western blot test was employed to verify the presence of the crucial proteins active p-AKT and β-catenin. Additionally, the cell indicator Na^+^/K^+^ ATPase was examined through immunofluorescence and Western blot techniques. These findings were then evaluated in comparison to those from standard induction protocols.

### 2.8 Animals

Twenty fit New Zealand White rabbits, both male and female, aged between 8–10 weeks and with a weight range of 2.0–4.0 kg, sourced from Xilingjiao Experimental Animal Breeding Center in Jinan, Shandong Province, China, were selected for penetrating keratoplasty procedures. Acellular porcine cornea matrices were prepared using fresh pig eyes from Jinan Welcome Food Co. Ltd., Jinan, China. These animal experiments were conducted at the Medical Animal Experimental Research Center of Qilu Hospital, Shandong University. All procedures adhered to the guidelines of the Institutional Animal Care of the Medical Animal Experimental Research Center of Qilu Hospital, Shandong University, China, and received approval from the Association for Laboratory Animal Care.

### 2.9 Tissue-engineered corneal preparation and assessment

The APCM was prepared as previously described (26). In a concise process, fresh pig eyes were initially rinsed with phosphate-buffered saline (PBS) that included 1% penicillin/streptomycin. Using a trephine, the central portions of the corneas, measuring 11 mm in diameter, were excised. These corneas were then submerged in 1.5 M sterile saline solution for 2 days, with the solution refreshed at the 24 h mark, followed by a 48 h incubation in a mixture of DNAse and RNAse (5 U/mL each, sourced from Sigma). Afterward, the tissues underwent a 72 h wash in sterile PBS enhanced with 1% penicillin/streptomycin, refreshing the solution every 24 h. This decellularization process took place at 4°C with constant stirring. The acellular porcine cornea matrix (APCM) was then shaped into a lamella of 400 μm thickness, incorporating Bowman’s membrane into the APCM scaffold (AS), following the fabrication process we previously established (20).

CEC-like cells were used to develop the TEC. AS was placed in an individual well of 24-well plates and soaked in a CEC-like cell growth medium at 37°C for 24 h. Subsequently, 3 × 10^5^ CEC-like cells in the 30 μL growth medium were gently seeded on the stromal side of the AS, allowing attachment for 2 h before completely immersing in the CEC-like cell culture medium. The TEC was cultured for 7 days. Prior to transplantation, the TEC was sectioned into grafts measuring 5 mm in diameter.

### 2.10 Penetrating corneal transplantation in rabbits

To assess the functionality and biocompatibility of the construct, penetrating keratoplasty was conducted. Using the Excel method for random assignment, twenty rabbits were evenly split into two categories: an experimental category and a control category, with ten rabbits in each. To minimize potential confounders, two groups were conducted on the same day and the order was randomly. Throughout the various phases of the experiment, only the corresponding author had knowledge of the group assignments. Each rabbit in the experimental group underwent transplantation with TEC, and each rabbit in the control group underwent transplantation with AS. In summary, the rabbits underwent intravenous anesthesia using 3% pentobarbital sodium for systemic anesthesia and 0.4% oxybuprocaine hydrochloride for local anesthesia. A 4.5 mm trephine and corneal scissors were employed to excise the recipient’s cornea, either from the left or right eye. The anterior chamber was then filled with a viscoelastic substance (Alcon). The grafts that had been prepared were attached to the recipient site using 10-0 nylon stitches (Alcon). Following the procedure, injections around the eyeball and under the conjunctiva of triamcinolone and dexamethasone were given. Post-surgery, treatments with tobramycin, dexamethasone, and cyclosporine A were administered three to four times each day. Each operated eye was documented using a slit-lamp microscope. Fluorescein dye was applied to measure the rate of re-epithelialization. A Visante OCT device was used to ascertain the central corneal thickness, and intraocular pressure was gauged using a tonometer at the 1st, 2nd, and 3rd weeks post-operation.

### 2.11 Histopathology

CEC-like cells on the TEC were enumerated utilizing optical microscopy following dual coloring with alizarin red S and trypan blue (from Solarbio). The TEC was rinsed gently with PBS, and 0.4% Trypan blue dye drops were placed on the CEC-like cells for staining for approximately 3 min. After rinsing with PBS, 0.2% alizarin red S dye drops were placed on the CEC-like cells for approximately 2 min. The staining was observed under a microscope. Three view fields (400×) were examined for each sample.

For histological examination, both the AS and TEC were fixed in 4% paraformaldehyde and processed for embedding in both paraffin-embedded and frozen sections. Slices were then prepared and subjected to hematoxylin and eosin (HE) staining as well as DAPI staining following standard procedures. Three weeks post-transplantation, rabbits were euthanized through intravenous administration of an overdose of pentobarbital sodium, and their postoperative eyes were extracted. Half of the corneas were preserved by freezing and embedding in an OCT compound, while the other half were fixed in 4% formaldehyde and encased in paraffin. Sections measuring 5 μm thick were cut and underwent immunofluorescent labeling and HE coloring using conventional methods.

### 2.12 Statistical analyses

Every test was replicated independently at least three times, and the outcomes were depicted as means ± SEM. Statistical analysis was conducted using one-way ANOVA through SPSS v24.0 software. Image manipulation and data visualization were executed utilizing GraphPad Prism 8.0.2 and Adobe Photoshop CC 20.0.5 software. A significance threshold of *p* < 0.05 was utilized to ascertain statistical significance.

## 3 Results

### 3.1 Differentiation of CEC-like cells from SKPs

**Figure 1:**
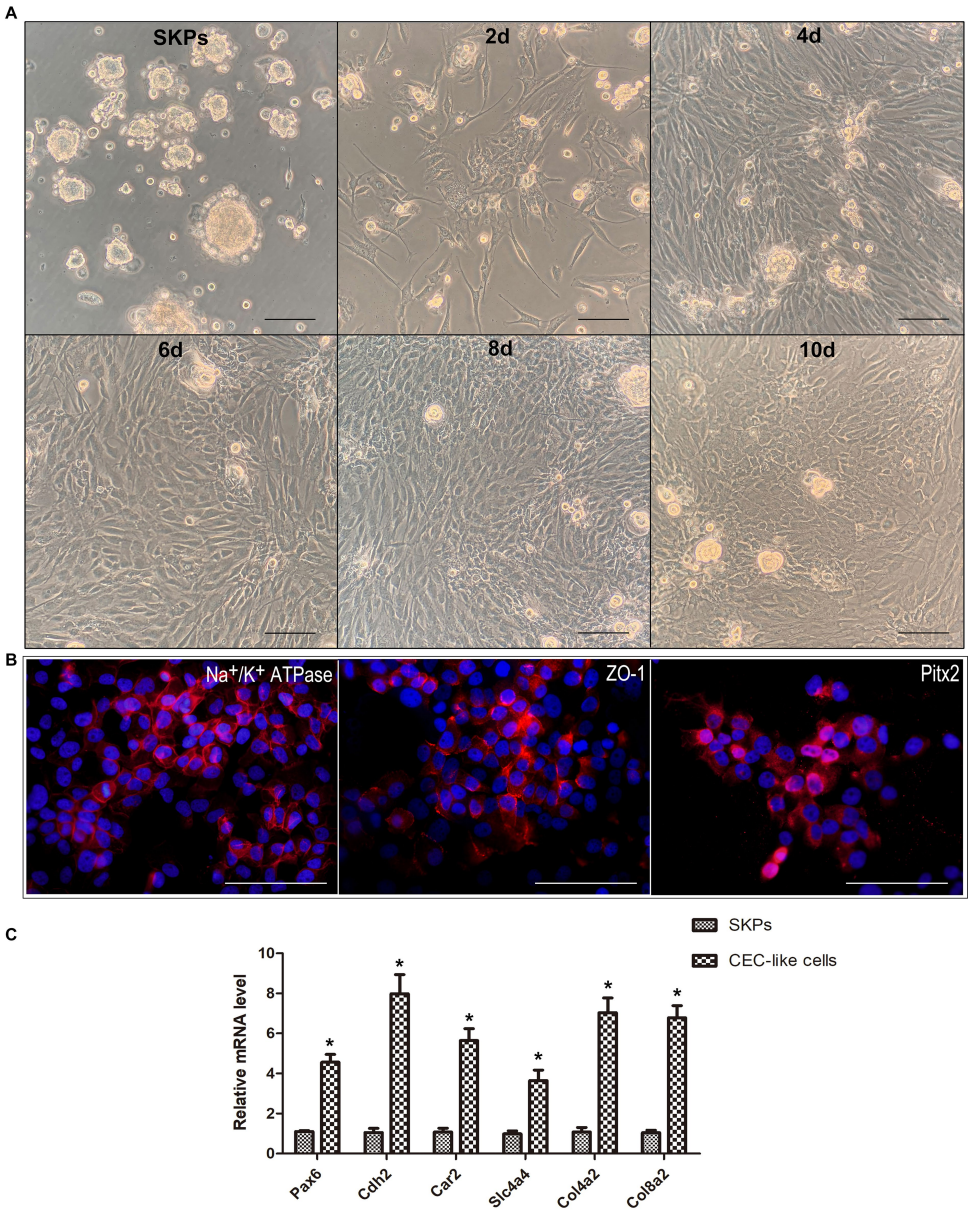
SKPs differentiated into CEC-like cells. **(A)** SKPs were cultured as floating spheres. During cell differentiation, the morphology of the cells changed gradually. On day 10, the cells became the most endothelial-like and formed a mosaic monolayer. **(B)** Immunofluorescence showed that the CEC-like cells expressed CEC markers Na^+^/K^+^ ATPase, ZO-1, and Pitx2 after 10 days of differentiation. **(C)** RT-PCR showed that the CEC-like cells expressed CEC markers Pax6, Cdh2, Car2, Slc4a4, Col4a2 and Col8a2. (Data are mean ± SEM, *p < 0.05, *n* = 3, scale bar = 100 μm).

SKPs were subjected to suspension culture for a duration of 2–3 weeks, resulting in the formation of floating spheres. Immunofluorescent staining demonstrated the expression of nestin, fibronectin, and vimentin in the SKPs (refer to Supplementary Figure S1). Following this, SKPs were dissociated into individual cells and then co-cultured with B4G12 cells in transwells to facilitate the generation of CEC-like cells. During cell differentiation, the cells showed elongated or irregular shapes after 2 days, and the shape tended to change. At 4 days, a significant increase in cells was observed, and some cells were polygonal. At 6 days, the cell morphology changed obviously, and a large number of polygonal endothelial-like cells appeared. Within 8 days, a majority of the cells had adopted an endothelium-like morphology, organizing into a mosaic monolayer. At day 10, the cells were more tightly connected and the endothelium-like morphology remained unchanged (Figure 1A). Immunofluorescent staining revealed the expression of Na^+^/K^+^ ATPase, ZO-1, and PITX2 by the CEC-like cells following 10 days of differentiation (Figure 1B). RT-PCR showed that CEC-like cells express significantly higher levels of other corneal endothelial markers including paired box 6 (Pax6), N-cadherin (Cdh2), carbonic anhydrase type 2 (Car2), solute carrier family 4 (Slc4a4), collagen type IV alpha 2 (Col4a2) and collagen type VIII alpha 2 (Col8a2) compared to SKPs (Figure 1C).

### 3.2 RNA-sequencing of SKPs and CEC-like cells

Expression variation test was employed to identify DEGs, whereby genes with an FDR < 0.05 and |log2FC| ≥ 1 were deemed significant. GO annotation analysis unveiled the top 20 GO categories enriched in DEGs, encompassing cellular component, biological process, and molecular function classifications. Furthermore, KEGG enrichment analysis was conducted to unveil the KEGG pathways significantly enriched with DEGs.

**Figure 2:**
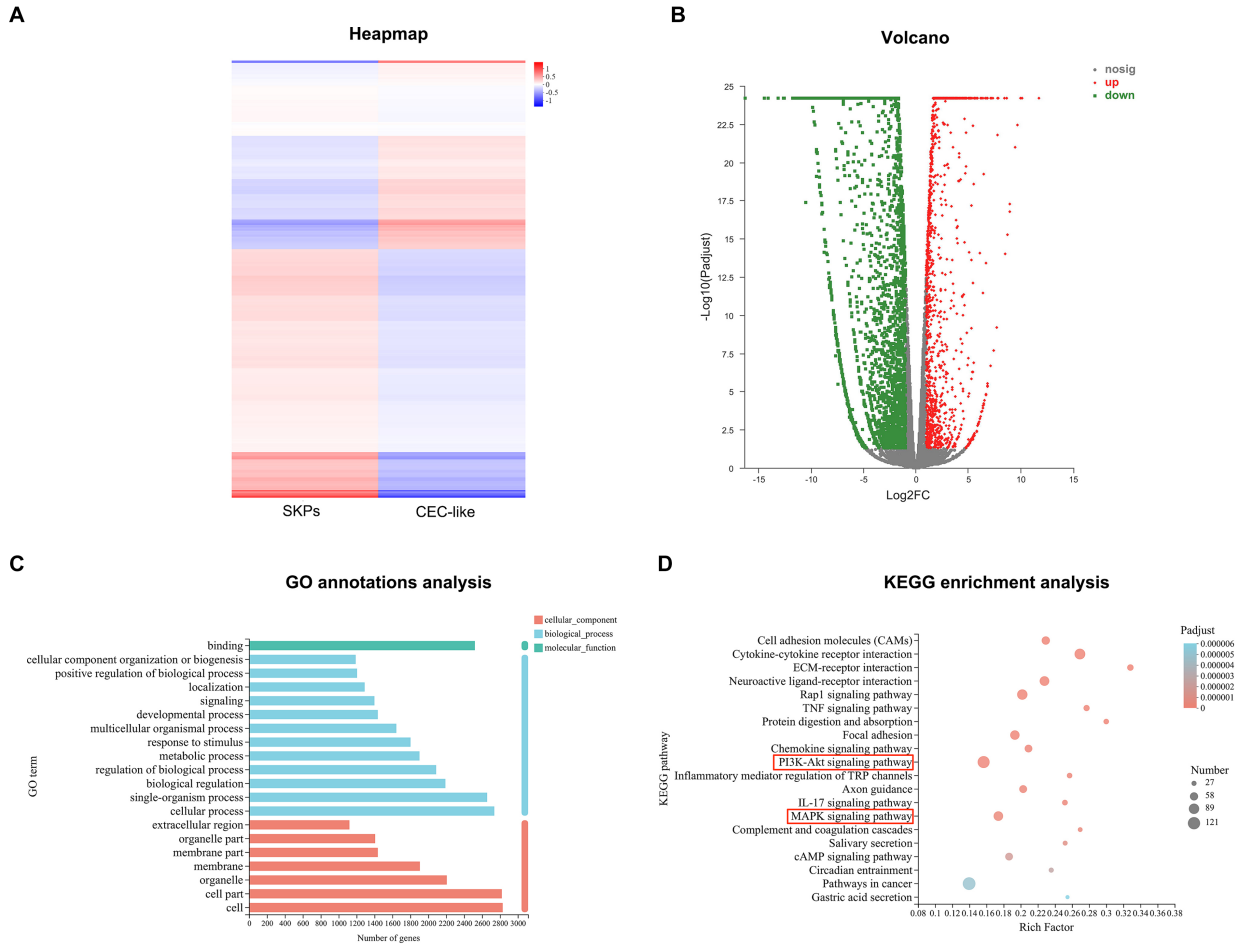
RNA-Sequencing of SKPs and CEC-like cells. **(A)** Heapmap of SKPs versus CEC-like cells. **(B)** Volcano plots of SKPs versus CEC-like cells. **(C)** GO annotation analysis of SKPs versus CEC-like cells. **(D)** KEGG enrichment analysis of SKPs versus CEC-like cells.

In total, 3,842 significant DEGs were recognized in both SKPs and CEC-like cells, comprising 1,235 upregulated genes and 2,607 downregulated genes (Figures 2A,B). GO annotation analysis revealed that the notable DEGs in SKPs and CEC-like cells were chiefly enriched in cellular elements, encompassing cells, cell parts, and organelles, alongside biological processes such as cellular activities, individual organism functions, and biological control. Moreover, the DEGs showed a significant association with molecular functions, particularly binding (Figure 2C). According to KEGG enrichment analysis, the predominant pathways associated with cell differentiation included cell adhesion molecules, cytokine-receptor signaling, ECM-receptor interaction, the PI3K/Akt signaling pathway, and the MAPK signaling pathway, among others (Figure 2D).

### 3.3 PI3K/Akt and MAPK/Erk pathways played important roles in cell differentiation

**Figure 3:**
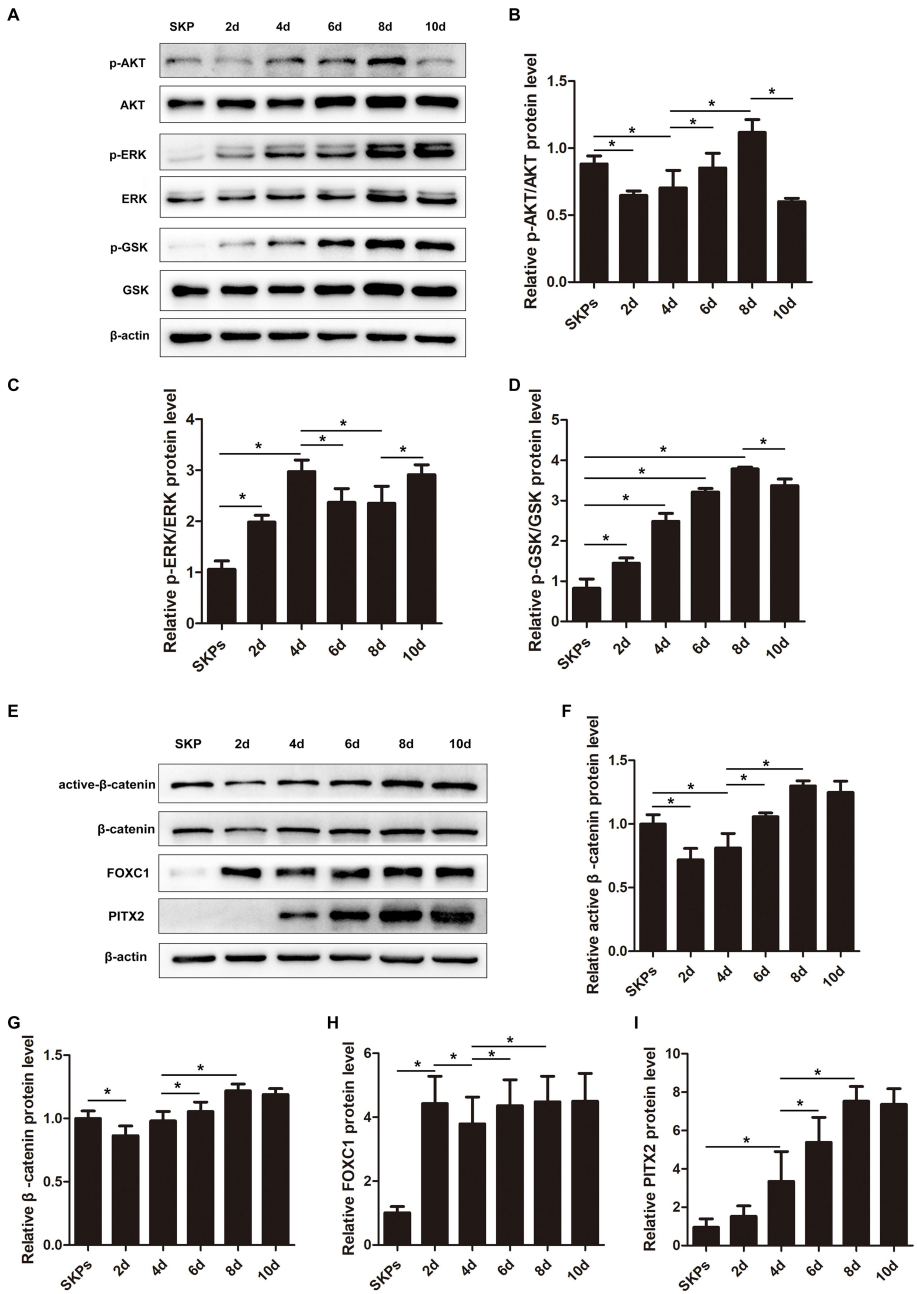
Pi3k/Akt, MAPK/Erk, WNT/β-catenin pathways, Pitx2, and Foxc1 factors played important roles during cell differentiation. **(A)** Western blotting showed the protein levels of major molecules in the pathway including P-AKT, AKT, p-ERK, ERK, p-GSK, and GSK. **(B–D)** Quantification of the relative protein levels in **(A)** is shown. **(E)** Western blotting showed the protein levels of active β-catenin, β-catenin, Foxc1, and Pitx2. **(F–I)** Quantification of the relative protein levels in **(E)** is shown. (Data are mean ± SEM, *p < 0.05, *n* = 3).

The PI3K/Akt and MAPK/Erk pathways, which are activated by RTK-mediated growth factor signaling, play crucial roles in the optimal generation and maturation of the NC. We examined the key molecules within these pathways. The findings implied that the expression of p-AKT was initially decreased on day 2, followed by an upregulation from days 4 to 8. However, by day 10, its expression dramatically decreased once more (Figures 3A,B). The p-ERK was upregulated during days 2–4 but was downregulated from day 6 before upregulation on day 8–10 (Figures 3A,C). The p-GSK was downstream of p-AKT and p-ERK, and it was upregulated gradually from days 2–8 but was downregulated on day 10 (Figures 3A,D).

### 3.4 WNT/β-catenin pathway, Foxc1, and Pitx2 controlled each other in cell differentiation

The WNT signaling is crucial in embryonic development and is crucial for sustaining embryonic eye development. In our prior investigation, we observed stimulation of the WNT/β-catenin pathway on day 8. In this present investigation, we noted a decrease in the expression of both active and total β-catenin initially, followed by a gradual increase (Figures 3E–G).

Additionally, we assessed the expression of Pitx2 and Foxc1, pivotal transcription factors linked to ocular advancement. The expression of Foxc1 exhibited a notable increase on day 2, followed by a slight decrease on day 4; however, it subsequently increased and maintained stability after day 6 (Figures 3E,H). Unlike Foxc1, the expression of Pitx2 continued to increase; it peaked on day 8 and remained stable (Figures 3E,I).

### 3.5 Inhibition of PI3K/Akt and WNT/β-catenin pathways blocks cell differentiation

**Figure 4:**
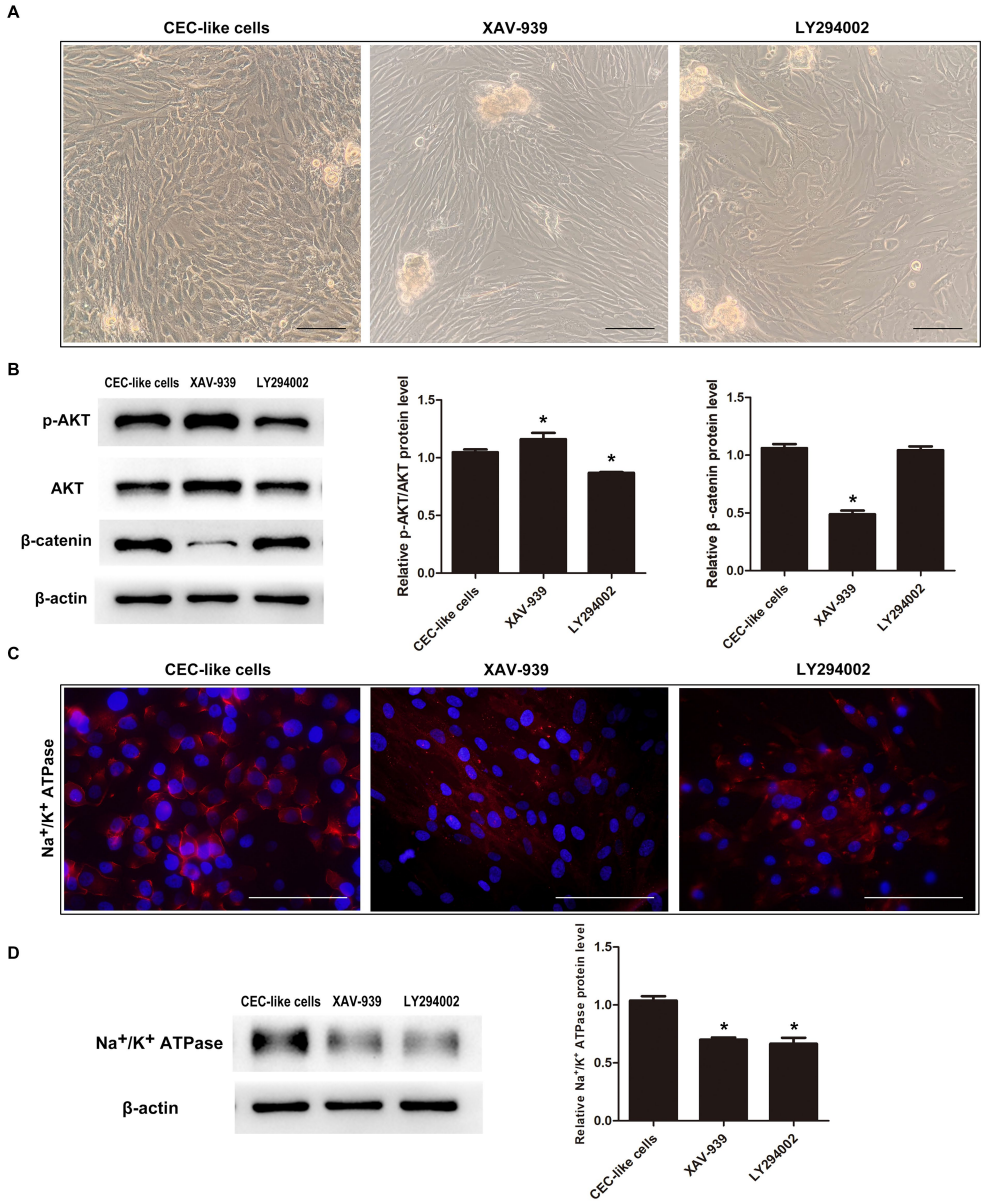
The relationship between the PI3K/Akt and WNT/β-catenin pathways during cell differentiation. **(A)** When the WNT/β-catenin pathway inhibitor XAV-939 or PI3K/Akt pathway inhibitor LY294002 were added to the differentiation system, the cells could not transform into CEC-like cells. **(B)** Western blotting showed that the two pathways were interrelated. **(C)** Immunofluorescence showed that the cells in the XAV-939 group and LY294002 group expressed extremely low levels of Na^+^/K^+^ ATPase. **(D)** Western blotting showed that the relative Na^+^/K^+^ ATPase protein levels in the two groups were downregulated relative to the CEC-like cells. (Data are mean ± SEM, *p < 0.05, *n* = 3, scale bar = 100 μm).

Given the interplay and functions of PI3K/Akt and WNT/β-catenin signaling in lineage commitment, we inhibited these pathways to observe their impact on cell differentiation. Upon addition of the WNT/β-catenin pathway inhibitor XAV-939 to the differentiation mechanism, there was a reduction in β-catenin levels alongside an increase in p-AKT levels. Conversely, when the PI3K/Akt pathway inhibitor LY294002 was introduced, p-AKT levels decreased while β-catenin levels remained unchanged (Figure 4B). Cells in the XAV-939 category displayed a spindle-like structure surrounding spheres, whereas those in the LY294002 group displayed irregular morphology with polygonal cells (Figure 4A). Immunofluorescence analysis indicated markedly reduced expression levels of Na^+^/K^+^ ATPase in cells from both groups (Figure 4C). Western blotting further corroborated these findings, revealing a downregulation in the protein expression levels of Na^+^/K^+^ ATPase in both groups compared to the normal differentiation group (Figure 4D).

### 3.6 CEC-like cells grew well on the APCM scaffold

**Figure 5:**
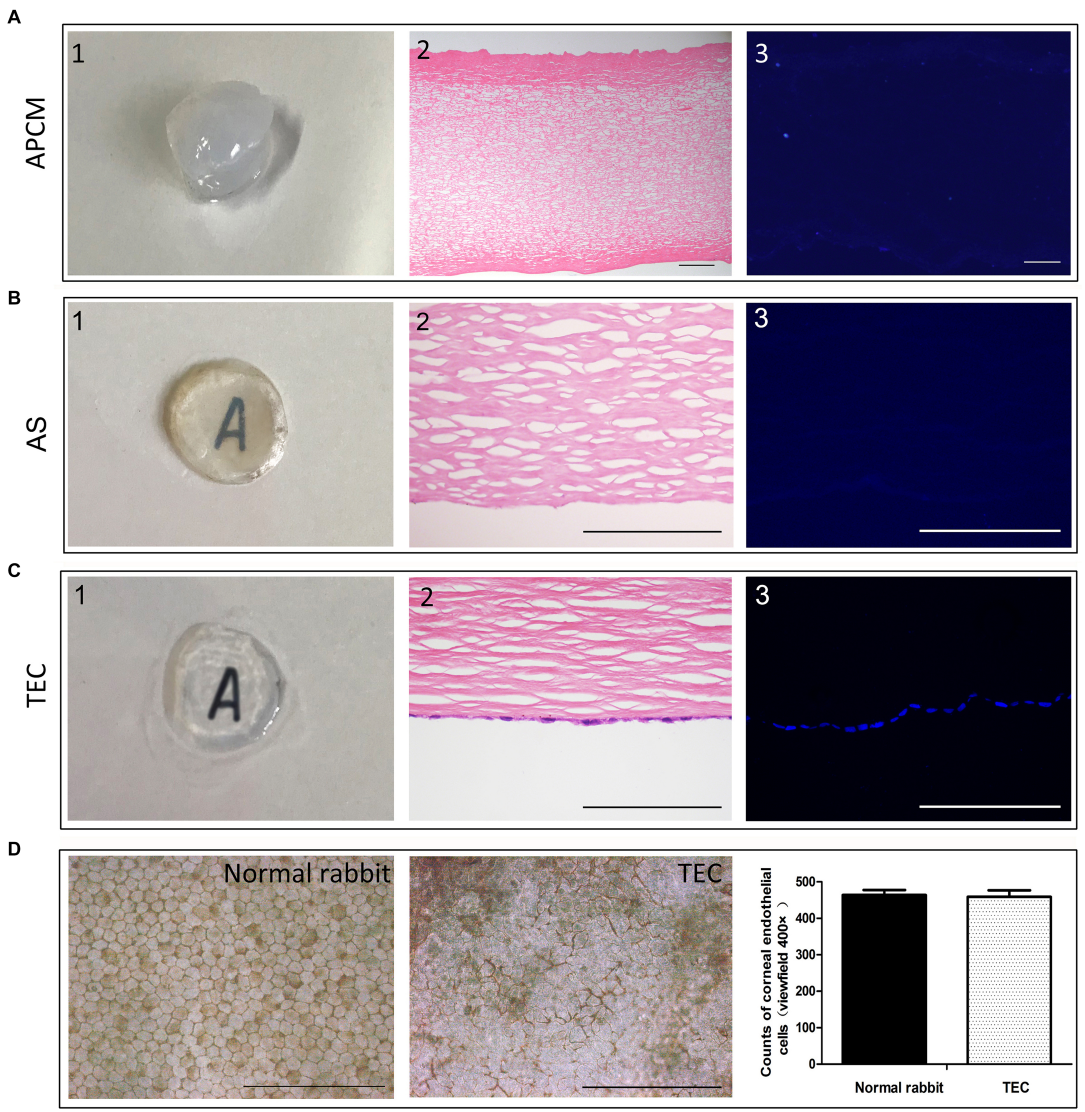
Development of the tissue-engineered cornea. **(A)** The APCM was quite opaque and thick. HE and DAPI staining showed that the cells were removed completely. **(B)** The AS showed good optical transparency. HE showed that the cutting surface was very smooth. **(C)** TEC was well-developed, and the CEC-like cells formed a monolayer attached tightly to the stromal cutting side. **(D)** Alizarin red and trypan blue staining showed that the monolayer polygonal CEC-like cells were alive and closely connected in the TEC. The number of CEC-like cells was similar to that of the cells in the native rabbit corneal endothelium. (Data are mean ± SEM, *p < 0.05, *n* = 3, scale bar = 100 μm).

Upon gross observation, the acellular porcine cornea matrix (APCM) appeared notably opaque and thick following the decellularization procedure. Histological examinations via HE and DAPI staining revealed the thorough elimination of cells. The integrity of the Bowman’s membrane remained intact, and the organization of collagen fibers seemed structured, underscoring the preservation of the organic corneal structure throughout the decellularization process (Figure 5A). The APCM scaffold (AS) showed good optical transparency because of water outflow during the cutting process and the under letter could be seen. HE staining showed that the cutting surface was very smooth, providing a good adhesion surface for cells (Figure 5B). The tissue-engineered cornea (TEC) exhibited well, with the CEC-like cells forming a tightly adherent monolayer on the stromal cutting side (Figure 5C). Coloring with alizarin red and trypan blue indicated the presence of viable, closely interconnected polygonal CEC-like cells within the monolayer of the TEC. Cell counting analysis revealed that the quantity of CEC-like cells closely resembled that of cells in the native rabbit corneal endothelium (Figure 5D).

### 3.7 Tissue-engineered cornea performed good function *in vivo*

**Figure 6:**
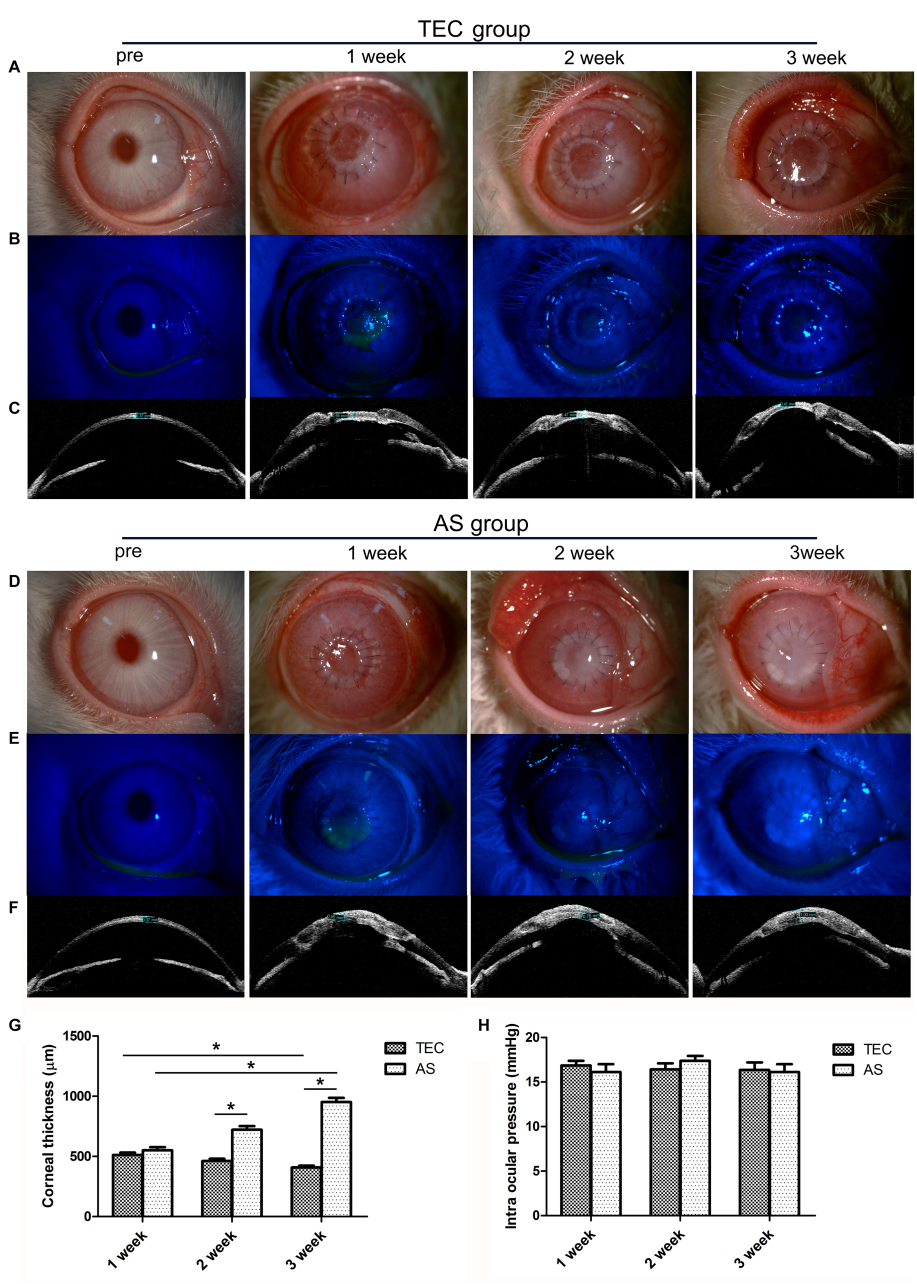
Clinical observation of TEC and AS transplantation in rabbits. **(A)** Slit-lamp photograph shows that the graft in the TEC group was initially cloudy at first but became transparent gradually. **(B)** Fluorescein staining showed that the graft in the TEC group was completely re-epithelialized after 3 weeks. **(C)** OCT showed that the graft thickness in group TEC decreased gradually. **(D)** Slit-lamp photograph shows that the graft in the AS group was more opaque; the graft was completely opaque at 3 weeks. **(E)** Fluorescein staining showed that the graft in the AS group was completely re-epithelialized at 3 weeks. **(F)** OCT showed that the graft thickness of the AS group increased gradually. **(G)** The graft thickness of the TEC and AS groups. **(H)** The intraocular pressure of the TEC and AS groups. (Data are mean ± SEM, *p < 0.05, *n* = 3, scale bar = 100 μm).

In the TEC group, the grafts were initially cloudy, and it became transparent gradually. At the 3 week mark, the pupil and iris were discernible through the tissue-engineered cornea (TEC) (refer to Figure 6A). In the AS group, the grafts became more opaque, and it was completely opaque at 3 weeks (Figure 6D). There was a notable discrepancy in transparency between the two groups. Additionally, none of the grafts in either group had undergone epithelialization at the time of transplantation. During week 1, however, the grafts were re-epithelialized with coverage of approximately 50%. The grafts were completely re-epithelialized after 3 weeks, and the corneal fluorescein staining was negative (Figures 6B,E).

During week 1, OCT imaging revealed no substantial variance in corneal thickness between the TEC and AS groups (*p* > 0.05). However, the thickness for the TEC group decreased gradually, while that of the AS group increased gradually. After 3 weeks, the thicknesses for the two groups were averaged at 408 μm and 952 μm respectively, which were significantly different (*p* < 0.05) (Figures 6C,F,G). Prior to surgery, the mean intraocular pressure stood at 16.07 ± 1.1 mmHg (mean ± SD). Subsequent to the surgery, there was no discernible elevation in intraocular pressure, and it remained within the normal range for both groups throughout the follow-up period (*p* > 0.05) (refer to Figure 6H).

After surgery, there was some fibrinoid exudation in the anterior chamber. The exudation gradually decreased, and there were no new keratic precipitates, and aqueous flare and neovascularization appeared during the observation period. We observed mild graft degradation, but it gradually resolved.

### 3.8 Histological observation after transplantation for 3 weeks

**Figure 7:**
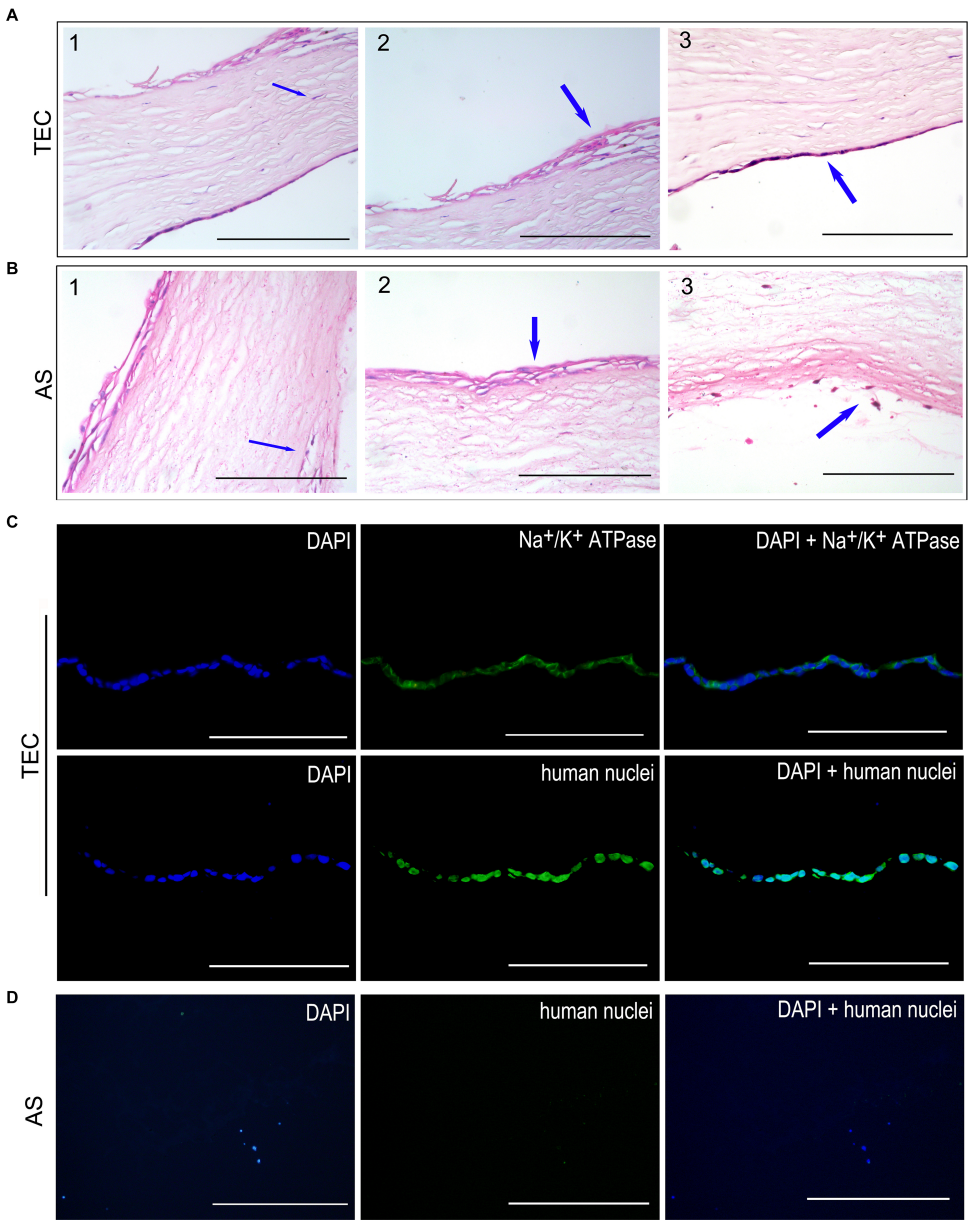
Histological observation after transplantation for 3 weeks. **(A)** HE staining of TEC group. The CEC-like cells formed a monolayer well attached to the stromal side. The host keratocytes migrated into the grafts. The restored epithelium consisted of 2–4 layers of cells well-attached to Bowman’s membrane. **(B)** HE staining of the AS group. No endothelial cells were detected on the stromal side. A few inflammatory cells were present in the stroma and edge of the graft. **(C)** The immunofluorescent staining showed that the CEC-like cells expressed Na^+^/K^+^ ATPase and human nuclei in the TEC graft. **(D)** There were no endothelial cells in the AS group (scale bar = 100 μm).

In the TEC group, the CEC-like cells developed a cohesive monolayer firmly adhered to the stromal side of the TEC (Figure 7A3). Conversely, no endothelial cells were identified on the stromal side of the AS group (Figure 7B3). Notably, keratocytes from the host corneal stroma had moved into regions of the grafts in both groups (Figures 7A1,B1). Moreover, the regenerated epithelium comprised 2–4 layers of cells securely adhered to the Bowman’s membrane in both groups (Figures 7A2,B2). A limited quantity of inflammatory cells were observed within the stroma and at the periphery of the graft (Figure 7B3).

Immunofluorescent staining of human nuclei verified that the endothelium in the TEC group consisted of seeded CEC-like cells rather than being a result of host endothelial migration. Notably, the CEC-like cells retained expression of Na^+^/K^+^ ATPase, signifying the preservation of pump function *in vivo* post-transplantation (Figure 7C). Conversely, endothelial cells were absent in the AS group (Figure 7D).

## 4 Discussion

SKPs have been effectively transformed into CEC-like cells, exhibiting morphology and traits akin to those of typical human CECs. The co-culturing method alongside B4G12-HCEC cells proves to be straightforward and productive. Such advancements underscore the potential promise of cell-mediated therapy employing CEC-like cells for addressing impaired corneal endothelial function. Nonetheless, the precise molecular mechanisms governing the differentiation of CEC-like cells from SKPs remain ambiguous (27). This study primarily focused on investigating the molecular mechanisms underlying cell differentiation. Transcriptome sequencing of SKPs and CEC-like cells revealed significant differential expression in 3842 genes. GO annotation analysis indicated that gene expression altered the classification of cellular components, biological processes, and molecular function, suggesting that the cell completely changed during cell differentiation. The KEGG enrichment analysis unveiled a pathway crucial for the morphological and functional transitions from SKPs to CEC-like cells. Activation of pathways such as molecular interactions in cell adhesion, ECM recognition, and cytokine signaling suggested that the differentiated CEC-like cells from SKPs acquired adhesive properties and developed tight junctions. Additionally, the PI3K/Akt and MAPK signaling pathways exhibited activation, each exerting significant roles in NC specification, proliferation, survival, migration, and differentiation (28, 29). The WNT pathway is not enriched in the KEGG enrichment analysis; nevertheless, it is crucial in maintaining embryonic eye progression and formation. This study delved into a detailed examination of the PI3K/Akt, MAPK, and WNT pathways.

Both the PI3K/Akt and MAPK/Erk signaling pathways played pivotal roles in governing cell growth and differentiation. Activation of these pathways could be triggered by a variety of receptors, encompassing RTKs (such as PDGFRs, FGFRs, EGFRs, and insulin receptors), GPCRs, and cytokine receptors, among others (30). We observed fluctuations in the expressions of p-AKT and p-ERK during cell differentiation. p-AKT was suppressed when p-ERK was activated and vice versa. The reciprocal regulation between the MAPK/Erk and PI3K/Akt pathways has been well-established. Vasudevan noted heightened AKT activation upon MEK1/2 suppression and elevated ERK1/2 phosphorylation reacting to PI3K inhibition in neural crest-derived mouse embryonic palatal mesenchyme cells (31). Carole demonstrated that MAPK activation is conspicuous in blastula stem cells before they transition from the pluripotent state, with a subsequent decline as cells become lineage-restricted. Conversely, PI3K/Akt signaling is minimal in pluripotent cells, but it experiences a substantial upsurge as cells undergo lineage restriction (32). The interaction between these two pathways is widely recognized to be heavily reliant on context and can exhibit either cooperative or antagonistic behavior. GSK3β is downstream of AKT and ERK, which can deactivate GSK3β activity by phosphorylation (33). In our experiment, the p-GSK3β was initially upregulated before it became downregulated after day 8. It seems that the activity of GSK3β is a result of the collaboration between AKT and ERK or RTK signaling. Hence, the activation of RTK signaling pathways might play a pivotal role in cellular differentiation.

The WNT signaling pathway is critical in embryonic progression and disease pathogenesis. Throughout embryonic development, WNT signaling not only contributes to the establishment of the dorsoventral axis but also engages in numerous stages of development, like the formation of cell polarity and determination of cell fate (34, 35). Within the eye, the WNT signaling pathway is intricately linked with the progression of various structures, including the embryonic eye field, cornea, lens, retina, and blood vessels (36). Nonetheless, its role in corneal endothelium development remains ambiguous. The WNT/β-catenin signaling pathway encompasses classical WNT signaling, with β-catenin serving as the central component. Upon activation by WNTs, the phosphorylation and deactivation of GSK3β occur. As a result, β-catenin accumulates and relocates to the nuclei. Within the nucleus, β-catenin interfaces with the TCF/LEF family of gene regulators, thereby modulating the expression of their target genes (37). During cell differentiation, p-AKT/AKT and β-catenin were both downregulated on day 2 but subsequently upregulated from days 4–8, indicating their connection. Taking into account their interrelation and significance in lineage restriction, we selectively suppressed PI3K/Akt and WNT/β-catenin signaling to assess their impact on cell differentiation. The outcomes revealed that inhibiting these pathways impeded cell differentiation, leading to a reduction in Na^+^/K^+^ ATPase expression. The inhibition of β-catenin increased p-AKT/AKT, indicating negative feedback between the two pathways, suggesting that PI3K/Akt signaling was upstream of β-catenin. Nevertheless, the inhibition of PI3K did not significantly downregulate β-catenin, indicating that without PI3K signaling there were other signals directly affecting the WNT/β-catenin pathway in the process of cell specialization.

The WNT/β-catenin signaling pathway exhibits intricate interplay with retinoic acid, Pitx2, and Foxc1 (38, 39). The retinoic acid signaling originating from the optic cup, lens, and surface ectoderm crest is pivotal in coordinating the morphogenesis of the anterior segment. This signaling cascade activates the expression of crucial transcription factor genes within the neural crest, thereby regulating its development (40). Pitx2 and Foxc1 are required for anterior segment development (41, 42). Their loss results in agenesis or severe disruption of normal ocular structures. The expression of Pitx2 and Foxc1 in the periocular mesenchyme relies on the signaling of retinoic acid (43). Aberrant control of the WNT/β-catenin signaling pathway causes various ocular abnormalities attributed to disruptions in cell fate determination and specialization processes. Gage proposed that while intact canonical WNT signaling may not be necessary for neural crest migration to the eye primordia, it appears redundant for the initial formation of the eye during early morphogenesis, originating from both neural and surface ectoderm. He illustrated that while canonical WNT signaling is initially unnecessary for Pitx2 activation in ocular neural crest, it becomes crucial later on to sustain Pitx2 expression throughout anterior segment development (38). Skerjanc discovered that excessive expression of activated β-catenin resulted in enhancement of Foxc1 expression during cell differentiation (44). In our investigation, we assessed the expression levels of Pitx2 and Foxc1. Our findings indicated that Foxc1 expression notably surged on day 2 before experiencing a decline, followed by a gradual rise. Conversely, Pitx2 expression exhibited a substantial increase on day 4, followed by a steady upward trend. We inferred that the early stage of cell differentiation simulated the ocular neural crest stage, and retinoic acid played a critical role. Foxc1 and Pitx2 were activated by retinoic acid and Foxc1 earlier. Later in the morphogenesis of the corneal endothelium, the WNT/β-catenin signaling came into play, and Pitx2 expression was maintained. The relationship between Foxc1 and β-catenin was not clear, and we speculated that the decrease in Foxc1 levels was linked with WNT/β-catenin signaling fluctuations. Hence, Pitx2 and Foxc1 appeared to undergo precise temporal and spatial regulation linked to WNT signaling throughout the cell specialization process of SKPs into CEC-like cells.

**Figure 8:**
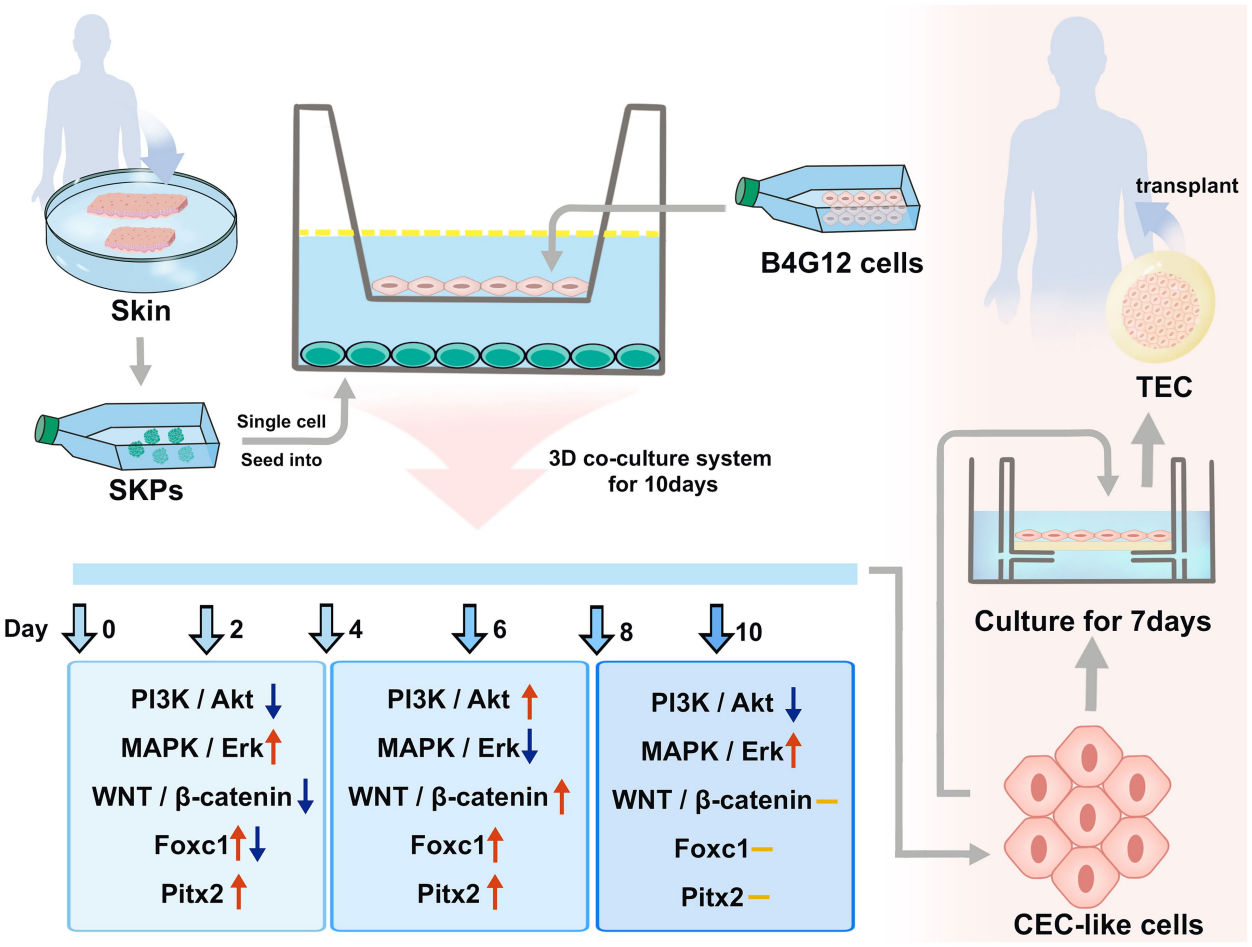
The differentiation of CEC-like cells from SKPs and the application of CEC-like cells.

The CECs developmental molecular regulation has not been established, and this may be attributed to the difficulty in distinguishing the endothelium from the cornea during embryonic eye development. SKPs were identified as precursors related to the embryonic neural crest and exhibited various characteristics similar to those of NC stem cells (7). Combining our data with previous reports, we speculated that our cell differentiation process mimicked the *in vitro* development of CECs (Figure 8). The cell differentiation was significantly influenced by the PI3K/Akt, MAPK/Erk, and WNT/β-catenin pathways, along with the participation of Pitx2 and Foxc1 factors. Crosstalk and connections exist between the pathways and factors. Further investigation is required to elucidate the intricate interplay among these pathways, considering their temporal, spatial, and cell-type-specific complexities. Therefore, our cell differentiation may provide an excellent and simple model for studying CECs development *in vitro*. It may also provide pre-organoid models for studying genetic corneal diseases. This is an important breakthrough.

To delve deeper into the functionality and potential applications of CEC-like cells, we embarked on developing a tissue-engineered cornea incorporating these cells. In an earlier study, we pioneered a fabrication method for sectioning APCM and validated its capability to sustain the viability of limbal epithelium-mimicking cells and cells resembling corneal endothelial cells originating from human embryonic stem cells (20). Thus, we used our CEC-like cells combined with an APCM scaffold to develop a tissue-engineered cornea. However, the TEC we constructed had only CEC-like cells but no keratocytes and epithelium. The reason was that corneal limbal stem cells could proliferate to produce epithelial cells (45). It has been reported in the literature that host keratocytes can migrate to the TEC graft after the TEC is transplanted to the host (46). We developed the de-epithelialized TEC to explore if they could function *in vivo*.

When we developed the TEC, we seeded cells in very few media and added media 2 h later to prevent media outflow from the AS and guarantee enough cell adhesion. The cell density was high; however, the endothelial contact inhibition characteristics could prevent the formation of multiple layer cells. Normally, endothelial cells grow on Descemet’s membrane, but the posterior lamellar of our scaffold was cut. Hence, the CEC-like cells were exclusively seeded on the stromal side. Findings indicated well growth of polygonal CEC-like cells on the stromal side of the TEC, forming a tightly connected mosaic monolayer. The quantity of CEC-like cells closely mirrored that of cells in the native rabbit corneal endothelium. We considered that APCM was cut with an equal force resulting in the trimmed stromal section that provided the surface for cell growth. Thus, the CEC-like cells grew well on the APCM scaffold and we successfully constructed the TEC.

Rabbit penetration corneal transplantation was performed with TEC or AS. We observed that corneas in the TEC group became transparent and corneal thickness decreased gradually. However, corneas in the AS group became more thick and opaque. Histological examination implied that the CEC-like cells maintained expressions of Na^+^/K^+^ ATPase. Meanwhile, the expression of human nuclei protein in the TEC group confirmed the source of endothelial cells; they were seeded CEC-like cells that were not from host endothelial cell migration. There were no endothelial cells on the stromal side in the AS group. We also confirmed that the host keratocytes can migrate into grafts as reported in the literature. Given that human keratocytes can hardly maintain their phenotype (47) and are uniformly distributed in TEC, we believed it was a good choice to develop TEC with no keratocytes. Corneal fluorescein staining and histological examination showed that host epithelial cells migrated and covered the graft completely within a few weeks. Our approach omitted the complicated process of seeding epithelial cells on TEC. However, the covered epithelium layers were few and fell off easily. When we prolonged the duration of observation, some TEC grafts were perforated. This may have been due to the stromal degradation during earlier stage because there was no epithelium coverage and inflammatory cell infiltration. In future studies, we can cover the ocular surface with an amniotic membrane to facilitate epithelial growth and prevent corneal perforation. As a result, CEC-like cells could be used to develop the tissue-engineered cornea and performed good function *in vivo*. This extends the utilization of CEC-like cells.

## 5 Conclusion

In summary, we differentiated SKPs into CEC-like cells with a 3D co-culture system by simulating the CEC developmental microenvironment and developed the tissue-engineered cornea with CEC-like cells. The involvement of the PI3K/Akt, MAPK/Erk, and WNT/β-catenin pathways, along with Pitx2 and Foxc1 factors, was notable throughout the differentiation process. Crosstalk and connections existed between the pathways and factors. The CEC-like cells grew well on the APCM scaffold, and the TEC graft performed well after transplantation to the rabbits. Our research provides experimental basis for CEC-like cell industrial production and drive the cells to be clinically applied in cellular replacement therapy or alternative graft substitution for treating corneal diseases in the future.

## Data Availability

The datasets presented in this study can be found in online repositories. The names of the repository/repositories and accession number(s) can be found in the article/Supplementary material.

## References

[1] Kinoshita S Koizumi N Ueno M Okumura N Imai K Tanaka H. Injection of cultured cells with a ROCK inhibitor for bullous keratopathy. N Engl J Med. (2018) 378:995–1003. doi: 10.1056/NEJMoa1712770, PMID: 29539291

[2] Price MO Mehta JS Jurkunas UV Price FW Jr. Corneal endothelial dysfunction: evolving understanding and treatment options. Prog Retin Eye Res. (2021) 82:100904. doi: 10.1016/j.preteyeres.2020.100904 32977001

[3] Gupta PK Berdahl JP Chan CC Rocha KM Yeu E Ayres B. The corneal endothelium: clinical review of endothelial cell health and function. J Cataract Refract Surg. (2021) 47:1218–26. doi: 10.1097/j.jcrs.0000000000000650, PMID: 34468459

[4] Catala P Thuret G Skottman H Mehta JS Parekh M Ni Dhubhghaill S. Approaches for corneal endothelium regenerative medicine. Prog Retin Eye Res. (2022) 87:100987. doi: 10.1016/j.preteyeres.2021.100987, PMID: 34237411

[5] Shen L Sun P Zhang C Yang L Du L Wu X. Therapy of corneal endothelial dysfunction with corneal endothelial cell-like cells derived from skin-derived precursors. Sci Rep. (2017) 7:13400. doi: 10.1038/s41598-017-13787-1, PMID: 29042661 PMC5645363

[6] Shen L Sun P Du L Zhu J Ju C Guo H. Long-term observation and sequencing analysis of SKPs-derived corneal endothelial cell-like cells for treating corneal endothelial dysfunction. Cell Transplant. (2021) 30:9636897211017830. doi: 10.1177/09636897211017830, PMID: 34053246 PMC8182626

[7] Fernandes KJ McKenzie IA Mill P Smith KM Akhavan M Barnabe-Heider F. A dermal niche for multipotent adult skin-derived precursor cells. Nat Cell Biol. (2004) 6:1082–93. doi: 10.1038/ncb1181, PMID: 15517002

[8] Walker H Akula M West-Mays JA. Corneal development: role of the periocular mesenchyme and bi-directional signaling. Exp Eye Res. (2020) 201:108231. doi: 10.1016/j.exer.2020.108231 33039457 PMC7942814

[9] Hatou S Shimmura S. Review: corneal endothelial cell derivation methods from ES/iPS cells. Inflamm Regen. (2019) 39:19. doi: 10.1186/s41232-019-0108-y, PMID: 31592286 PMC6775652

[10] Levis HJ Kureshi AK Massie I Morgan L Vernon AJ Daniels JT. Tissue engineering the cornea: the evolution of RAFT. J Funct Biomater. (2015) 6:50–65. doi: 10.3390/jfb6010050 25809689 PMC4384100

[11] Guerin LP Le-Bel G Desjardins P Couture C Gillard E Boisselier E. The human tissue-engineered cornea (hTEC): recent Progress. Int J Mol Sci. (2021) 22:31291. doi: 10.3390/ijms22031291, PMID: 33525484 PMC7865732

[12] Delaey J De Vos L Koppen C Dubruel P Van Vlierberghe S Van den Bogerd B. Tissue engineered scaffolds for corneal endothelial regeneration: a material's perspective. Biomater Sci. (2022) 10:2440–61. doi: 10.1039/d1bm02023d 35343525

[13] Wang S Ghezzi CE Gomes R Pollard RE Funderburgh JL Kaplan DL. In vitro 3D corneal tissue model with epithelium, stroma, and innervation. Biomaterials. (2017) 112:1–9. doi: 10.1016/j.biomaterials.2016.09.030 27741498 PMC5121002

[14] Yoshida J Yokoo S Oshikata-Miyazaki A Amano S Takezawa T Yamagami S. Transplantation of human corneal endothelial cells cultured on bio-engineered collagen Vitrigel in a rabbit model of corneal endothelial dysfunction. Curr Eye Res. (2017) 42:1420–5. doi: 10.1080/02713683.2017.1351568 28933958

[15] Peh GSL Ang HP Lwin CN Adnan K George BL Seah XY. Regulatory compliant tissue-engineered human corneal endothelial grafts restore corneal function of rabbits with bullous keratopathy. Sci Rep. (2017) 7:14149. doi: 10.1038/s41598-017-14723-z, PMID: 29074873 PMC5658403

[16] Kong B Sun W Chen G Tang S Li M Shao Z. Tissue-engineered cornea constructed with compressed collagen and laser-perforated electrospun mat. Sci Rep. (2017) 7:970. doi: 10.1038/s41598-017-01072-0, PMID: 28428541 PMC5430529

[17] Zhang J Zhang CW Du LQ Wu XY. Acellular porcine corneal matrix as a carrier scaffold for cultivating human corneal epithelial cells and fibroblasts in vitro. Int J Ophthalmol. (2016) 9:1–8. doi: 10.18240/ijo.2016.01.01, PMID: 26949602 PMC4768513

[18] Tsai MC Daniels JT. The impact of biomechanics on corneal endothelium tissue engineering. Exp Eye Res. (2021) 209:108690. doi: 10.1016/j.exer.2021.108690 34216616

[19] Wang F Song Q Du L Wu X. Development and characterization of an acellular porcine small intestine submucosa scaffold for use in corneal epithelium tissue engineering. Curr Eye Res. (2020) 45:134–43. doi: 10.1080/02713683.2019.1663386, PMID: 31514545

[20] Zhang C Du L Sun P Shen L Zhu J Pang K. Construction of tissue-engineered full-thickness cornea substitute using limbal epithelial cell-like and corneal endothelial cell-like cells derived from human embryonic stem cells. Biomaterials. (2017) 124:180–94. doi: 10.1016/j.biomaterials.2017.02.003, PMID: 28199886

[21] Pang K Du L Wu X. A rabbit anterior cornea replacement derived from acellular porcine cornea matrix, epithelial cells and keratocytes. Biomaterials. (2010) 31:7257–65. doi: 10.1016/j.biomaterials.2010.05.066, PMID: 20598368

[22] Pang K Du L Zhang K Dai C Ju C Zhu J. Three-dimensional construction of a rabbit anterior corneal replacement for lamellar keratoplasty. PLoS One. (2016) 11:e0168084. doi: 10.1371/journal.pone.0168084, PMID: 27930708 PMC5145227

[23] Biernaskie JA McKenzie IA Toma JG Miller FD. Isolation of skin-derived precursors (SKPs) and differentiation and enrichment of their Schwann cell progeny. Nat Protoc. (2006) 1:2803–12. doi: 10.1038/nprot.2006.422 17406538

[24] De Kock J Najar M Bolleyn J Al Battah F Rodrigues RM Buyl K. Mesoderm-derived stem cells: the link between the transcriptome and their differentiation potential. Stem Cells Dev. (2012) 21:3309–23. doi: 10.1089/scd.2011.0723, PMID: 22651824

[25] Gotze T Valtink M Nitschke M Gramm S Hanke T Engelmann K. Cultivation of an immortalized human corneal endothelial cell population and two distinct clonal subpopulations on thermo-responsive carriers. Graefes Arch Clin Exp Ophthalmol. (2008) 246:1575–83. doi: 10.1007/s00417-008-0904-6, PMID: 18696098

[26] Shafiq MA Gemeinhart RA Yue BY Djalilian AR. Decellularized human cornea for reconstructing the corneal epithelium and anterior stroma. Tissue Eng Part C Methods. (2012) 18:340–8. doi: 10.1089/ten.TEC.2011.0072, PMID: 22082039 PMC3338110

[27] Shen L Pan L Ju C Wu X. The role of Wnt/beta-catenin pathway for skin-derived precursors differentiating into corneal endothelial cell-like cells. Exp Eye Res. (2022) 218:109008. doi: 10.1016/j.exer.2022.109008 35219695

[28] Dinsmore CJ Soriano P. MAPK and PI3K signaling: at the crossroads of neural crest development. Dev Biol. (2018) 444:S79–97. doi: 10.1016/j.ydbio.2018.02.003, PMID: 29453943 PMC6092260

[29] Ciarlo C Kaufman CK Kinikoglu B Michael J Yang S Amato C. A chemical screen in zebrafish embryonic cells establishes that Akt activation is required for neural crest development. eLife. (2017) 6. doi: 10.7554/eLife.29145, PMID: 28832322 PMC5599238

[30] Hiratsuka T Bordeu I Pruessner G Watt FM. Regulation of ERK basal and pulsatile activity control proliferation and exit from the stem cell compartment in mammalian epidermis. Proc Natl Acad Sci USA. (2020) 117:17796–807. doi: 10.1073/pnas.2006965117 32651268 PMC7395546

[31] Vasudevan HN Mazot P He F Soriano P. Receptor tyrosine kinases modulate distinct transcriptional programs by differential usage of intracellular pathways. eLife. (2015) 4:7186. doi: 10.7554/eLife.07186, PMID: 25951516 PMC4450512

[32] Geary L LaBonne C. FGF mediated MAPK and PI3K/Akt signals make distinct contributions to pluripotency and the establishment of neural crest. eLife. (2018) 7:33845. doi: 10.7554/eLife.33845, PMID: 29350613 PMC5790379

[33] Tang X Li G Shi L Su F Qian M Liu Z. Combined intermittent fasting and ERK inhibition enhance the anti-tumor effects of chemotherapy via the GSK3beta-SIRT7 axis. Nat Commun. (2021) 12:5058. doi: 10.1038/s41467-021-25274-3, PMID: 34433808 PMC8387475

[34] Rim EY Clevers H Nusse R. The Wnt pathway: from signaling mechanisms to synthetic modulators. Annu Rev Biochem. (2022) 91:571–98. doi: 10.1146/annurev-biochem-040320-103615 35303793

[35] Steinhart Z Angers S. Wnt signaling in development and tissue homeostasis. Development. (2018) 145:146589. doi: 10.1242/dev.146589 29884654

[36] Fujimura N. WNT/beta-Catenin Signaling in Vertebrate Eye Development. Front Cell Dev Biol. (2016) 4:138. doi: 10.3389/fcell.2016.00138, PMID: 27965955 PMC5127792

[37] Colozza G Koo BK. Wnt/beta-catenin signaling: structure, assembly and endocytosis of the signalosome. Develop Growth Differ. (2021) 63:199–218. doi: 10.1111/dgd.12718 PMC8251975 33619734

[38] Zacharias AL Gage PJ. Canonical Wnt/beta-catenin signaling is required for maintenance but not activation of Pitx2 expression in neural crest during eye development. Dev Dyn. (2010) 239:3215–25. doi: 10.1002/dvdy.22459 20960542 PMC3073314

[39] Kumar S Duester G. Retinoic acid signaling in perioptic mesenchyme represses Wnt signaling via induction of Pitx2 and Dkk2. Dev Biol. (2010) 340:67–74. doi: 10.1016/j.ydbio.2010.01.027, PMID: 20122913 PMC2834877

[40] Kumar S Dolle P Ghyselinck NB Duester G. Endogenous retinoic acid signaling is required for maintenance and regeneration of cornea. Exp Eye Res. (2017) 154:190–5. doi: 10.1016/j.exer.2016.11.009 27840061 PMC5363406

[41] Gage PJ Kuang C Zacharias AL. The homeodomain transcription factor PITX2 is required for specifying correct cell fates and establishing angiogenic privilege in the developing cornea. Dev Dyn. (2014) 243:1391–400. doi: 10.1002/dvdy.24165 25044936 PMC4206698

[42] Lovatt M Yam GH Peh GS Colman A Dunn NR Mehta JS. Directed differentiation of periocular mesenchyme from human embryonic stem cells. Differentiation. (2018) 99:62–9. doi: 10.1016/j.diff.2017.11.003 29239730

[43] Seo S Chen L Liu W Zhao D Schultz KM Sasman A. Foxc1 and Foxc2 in the neural crest are required for ocular anterior segment development. Invest Ophthalmol Vis Sci. (2017) 58:1368–77. doi: 10.1167/iovs.16-21217, PMID: 28253399 PMC5361455

[44] Savage J Voronova A Mehta V Sendi-Mukasa F Skerjanc IS. Canonical Wnt signaling regulates Foxc1/2 expression in P19 cells. Differentiation. (2010) 79:31–40. doi: 10.1016/j.diff.2009.08.008 19782461

[45] Zhu J Wang LY Li CY Wu JY Zhang YT Pang KP. SPARC promotes self-renewal of limbal epithelial stem cells and ocular surface restoration through JNK and p38-MAPK signaling pathways. Stem Cells. (2020) 38:134–45. doi: 10.1002/stem.3100, PMID: 31644832

[46] Zhou Y Wu Z Ge J Wan P Li N Xiang P. Development and characterization of acellular porcine corneal matrix using sodium dodecylsulfate. Cornea. (2011) 30:73–82. doi: 10.1097/ICO.0b013e3181dc8184, PMID: 20861730

[47] Sidney LE Hopkinson A. Corneal keratocyte transition to mesenchymal stem cell phenotype and reversal using serum-free medium supplemented with fibroblast growth factor-2, transforming growth factor-beta3 and retinoic acid. J Tissue Eng Regen Med. (2016) 12:e203–15. doi: 10.1002/term.2316, PMID: 27685949

