# Supplementary figures and images for: Construction of tissue engineered cornea with skin-derived corneal endothelial-like cell and mechanism research for the cell differentiation
Source: Front Med (Lausanne). 2024 Sep 2;11:1448248. doi: 10.3389/fmed.2024.1448248 (PMC11402686; doi:10.3389/fmed.2024.1448248)

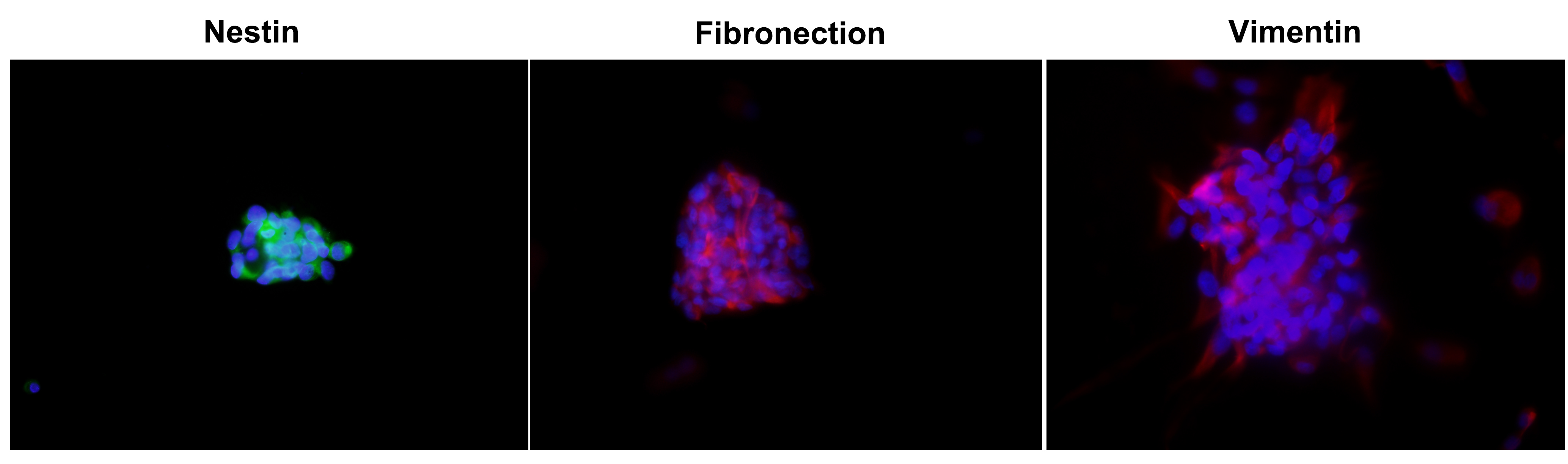

Supplement: Supplementary file 1 [file Image_1.JPEG]
